# Comparison of Fecal Microbiota of Horses Suffering from Atypical Myopathy and Healthy Co-Grazers

**DOI:** 10.3390/ani11020506

**Published:** 2021-02-15

**Authors:** Christina Wimmer-Scherr, Bernard Taminiau, Benoît Renaud, Gunther van Loon, Katrien Palmers, Dominique Votion, Hélène Amory, Georges Daube, Carla Cesarini

**Affiliations:** 1 Equine Clinical Department, Faculty of Veterinary Medicine, Bât. B41, Sart Tilman, University of Liège, 4000 Liège, Belgium; cwscherr@uliege.be (C.W.-S.); helene.amory@uliege.be (H.A.); 2 Fundamental and Applied Research for Animals & Health (FARAH), Faculty of Veterinary Medicine, Sart Tilman, University of Liège, 4000 Liège, Belgium; bernard.taminiau@uliege.be (B.T.); benoit.renaud@uliege.be (B.R.); dominique.votion@uliege.be (D.V.); georges.daube@uliege.be (G.D.); 3 Department of Food Sciences–Microbiology, Faculty of Veterinary Medicine, University of Liège, Avenue de Cureghem 10, Bât. B43b, 4000 Liège, Belgium; 4 Department of Functional Sciences, Pharmacology and Toxicology, Faculty of Veterinary Medicine, Bât. B41, Sart Tilman, University of Liège, 4000 Liège, Belgium; 5 Large Animal Internal Medicine, Gent University, 9820 Gent, Belgium; gunther.vanloon@ugent.be; 6 De Morette Equine Clinic, 1730 Asse, Belgium; katrien.palmers@demorette.be

**Keywords:** equine, fecal, intestinal, microbiome, rhabdomyolysis, intoxication, hypoglycin A, MCPA-CoA, Ruminococcaceae, Lachnospiraceae

## Abstract

**Simple Summary:**

Equine atypical myopathy is a muscular disease caused by a plant intoxication, which seems to affect only certain horses sharing a pasture, and the role of the intestinal bacteria (microbiota) in this selective impairment is still poorly understood. The aim of this study was to describe and compare fecal microbiota of horses suffering from atypical myopathy and healthy co-grazers. We concluded that fecal microbiota of horses suffering from atypical myopathy is different from their co-grazers and changes are more severe in horses that do not survive the disease. Understanding those changes may help in developing therapeutic and/or preventive strategies for horses at risk for atypical myopathy.

**Abstract:**

Equine atypical myopathy (AM) is caused by hypoglycin A (HGA) and methylenecyclopropylglycine (MCPG) intoxication resulting from the ingestion of seeds or seedlings of some *Acer* tree species. Interestingly, not all horses pasturing in the same toxic environment develop signs of the disease. In other species, it has been shown that the intestinal microbiota has an impact on digestion, metabolism, immune stimulation and protection from disease. The objective of this study was to characterize and compare fecal microbiota of horses suffering from AM and healthy co-grazers. Furthermore, potential differences in fecal microbiota regarding the outcome of diseased animals were assessed. This prospective observational study included 59 horses with AM (29 survivors and 30 non-survivors) referred to three Belgian equine hospitals and 26 clinically healthy co-grazers simultaneously sharing contaminated pastures during spring and autumn outbreak periods. Fresh fecal samples (rectal or within 30 min of defecation) were obtained from all horses and bacterial taxonomy profiling obtained by 16S amplicon sequencing was used to identify differentially distributed bacterial taxa between AM-affected horses and healthy co-grazers. Fecal microbial diversity and evenness were significantly (*p* < 0.001) higher in AM-affected horses as compared with their non-affected co-grazers. The relative abundance of families Ruminococcaceae, Christensenellaceae and Akkermansiaceae were higher (*p* ≤ 0.001) whereas those of the Lachnospiraceae (*p* = 0.0053), Bacteroidales (*p* < 0.0001) and Clostridiales (*p* = 0.0402) were lower in horses with AM, especially in those with a poor prognosis. While significant shifts were observed, it is still unclear whether they result from the disease or might be involved in the onset of disease pathogenesis.

## 1. Introduction

Outbreaks of equine atypical myopathy (AM) have occurred recurrently in Europe since the mid-1990s with more than 2500 cases reported in the last 10 years, mostly from central European countries [1,2]. The disease typically affects horses kept on pasture during spring/autumn, causing a non-exercise-induced acute rhabdomyolytic syndrome, affecting mainly postural and respiratory muscles and myocardium [2,3]. Clinical disease has been associated with hypoglycin A (HGA) intoxication resulting from the ingestion of seeds or seedlings of some *Acer* tree species [4] in Europe, mainly *Acer pseudoplatanus* (sycamore maple) [5]. Recently, it has been shown that methylenecyclopropylglycine (MCPG) is also involved in the development of the disease [6]. The clinical picture is characterized by the sudden onset of stiffness, muscular weakness, tachycardia and myoglobinuria, progressing in many cases to recumbency, respiratory difficulties and death [7,8] Despite the discovery of its etiology, there is still no cure for AM and symptomatic therapy is often unsuccessful [7,8,9]. Horses and ponies of any age and breed can be affected, and the reported mortality rate ranges from 43% to 97% [7,10].

It is the general consensus that intestinal absorption of the protoxins HGA and MCPG and their subsequent transformation to toxic metabolites are needed to develop AM, leading to rhabdomyolysis and, in many cases, even death [11]. Interestingly, and for a reason still unknown, not all horses pasturing in the same contaminated environment develop clinical signs of the disease. Published data show that HGA can be detected in the blood of clinically healthy horses pasturing in at-risk fields [5,12]. Although incidence of clinical disease has been linked to specific factors such as age, body condition, feeding and pasture management, the exact reason protecting certain horses from developing clinical signs remains largely unknown [5,12].

Studies in humans and animals have shown that intestinal microbiota has an impact on digestion, metabolism, immune stimulation and protection from pathogens and disease [13,14,15]. Research in human medicine increasingly confirms a link between dysbiosis of the gut microbiota and the pathogenesis not only of intestinal, but also of extra-intestinal, diseases [16]. Similarly, when compared with healthy or control horses, differences in the intestinal microbiome have been confirmed for various common equine illnesses, such as equine metabolic syndrome, carbohydrate-induced laminitis, colic, colitis and equine grass sickness. However, the significance of these findings is yet unclear [17,18,19,20,21,22]. To date, no published studies have investigated the potential role of intestinal microbiota in equine AM. 

A wide range of bacteria, including gut microbiota fermenters, are able to metabolize peptides and amino acids to use them for protein synthesis or the generation of metabolic energy [23,24]. Bacteria may also synthesize amino acids required for protein biosynthesis, creating a bilateral exchange between the gut microbiota and the host [25]. Branched-chain amino acids are metabolized in humans and in bacteria through the combined action of two enzymes, branched-chain amino acid aminotransferase (BCAT) and branched-chain oxoacid dehydrogenase (BCAD). Metabolism of MCPG and HGA to their active metabolites MCPF-CoA and MCPA-CoA is accomplished by the same two-step enzymatic system [24,26]. Specific intestinal bacteria containing the aforementioned enzymes could potentially contribute to the way HGA and MCPG are absorbed or metabolized in horses grazing contaminated pastures.

Our research hypothesis was that horses suffering from AM would have a different intestinal microbiota compared to their asymptomatic co-grazers. Horses showing clinical signs of AM could have a specific intestinal microbiome that contributes to or protects them from the development of disease. Decoding pathways of intoxication and/or identifying protective mechanisms against developing clinical disease could help in improving prevention and treatment of this devastating pathology. The objective of this study was to characterize and compare fecal microbiota of horses with AM and their healthy co-grazers. Furthermore, potential differences in fecal microbiota regarding the outcome of diseased animals were assessed. 

## 2. Materials and Methods 

### 2.1. Study Design and Sample Collection 

A prospective clinical study was conducted from autumn 2016 till spring 2019. The studied population included horses diagnosed with AM in three Belgian equine referral hospitals (i.e., Clinique Vétérinaire Universitaire de Liège, Faculty of Veterinary Medicine Ghent University, Dierenkliniek de Morette) during the study period. Whenever it was possible, for each sick horse, one or more healthy co-grazers were included in the study. Information about diet was collected retrospectively from the clinical records of hospitalized animals. 

A fresh fecal sample was obtained from each horse involved in the study. Sick horses were sampled at the time of admission to the clinic. Co-grazers were sampled at home, within 24 h since the first affected horse in the pasture showed clinical signs of AM. The 24 h limit was set in order to avoid the potential effect of management changes (i.e., dietary changes, being retired from pasture) implemented by the owner after confirmation of AM diagnosis to prevent disease in co-grazers. Fecal samples were collected in all cases directly from the rectal ampulla or from a pile of recently passed feces (<30 min). The core of a fecal ball was sampled in order to avoid external bacterial contamination [27] and immediately placed in a conservation milieu (Stool DNA stabilizer, PSP^®^ Spin Stool DNA Plus Kit 00310, Invitek, Berlin, Germany) and stored at −20 °C until total bacterial DNA extraction. 

### 2.2. Inclusion Criteria and Group Definition

Three separate groups were defined for the purpose of this study, referred to as atypical myopathy non-survivors (AM-NS), atypical myopathy survivors (AM-S) and healthy co-grazers (CG).

Diagnosis of AM was made based on:

- a tentative diagnosis of AM based on the algorithm proposed by van Galen et al. (2012) [7], i.e., a compatible history (non-exercise-induced acute rhabdomyolysis syndrome in a horse kept at pasture) and clinical signs highly suggestive of AM (i.e., acute onset of muscle weakness, stiffness and/or pigmenturia) during spring/autumn,
- elevated serum creatinine kinase activity, presence of HGA and MCPA-carnitine in serum and/or modified acylcarnitine profile compatible with the diagnosis of AM, when available [28,29].

Survivors were discharged from the clinic after a variable hospitalization period, whereas non-survivors died naturally from the disease during hospitalization or had to be euthanized due to significant clinical deterioration, continuous or unmanageable pain, prognostic or economic reasons. 

For the purpose of this study, a healthy co-grazer was defined as a horse:

- grazing in a pasture where a case of AM had been diagnosed in the previous 24 h.
- having a normal clinical and dynamic examination at walk (no signs of AM or other obvious disease) at the time of sampling.

### 2.3. Bacterial DNA Extraction and High-Throughput Sequencing

Total bacterial DNA was extracted from the stool samples with the PSP Spin Stool DNA Plus Kit 00310 (Invitek, Berlin, Germany), following the manufacturer’s recommendations. 

PCR amplification of the 16S rDNA V1–V3 hypervariable region and library preparation were performed with the following primers (with Illumina overhand adapters), forward (50-GAGAGTTTGATYMTGGCTCAG-30) and reverse (50-ACCGCGGCTGCTGGCAC-30). Each PCR product was purified with the Agencourt AMPure XP bead kit (Beckman Coulter, Pasadena, CA, USA) and submitted to a second PCR round for indexing, using Nextera XT index primers 1 and 2. After purification, PCR products were quantified using the Quant-IT PicoGreen (ThermoFisher Scientific; Waltham, MA, USA) and diluted to 10 ng/µL. A final quantification of each library was performed using the KAPA SYBR^®^ FAST qPCR Kit (KapaBiosystems; Wilmington, MA, USA) before normalization, pooling and sequencing on a MiSeq sequencer using V3 reagents (Illumina; San Diego, CA, USA). Positive controls using DNA from 20 defined bacterial species and negative controls (from extraction and PCR steps) were included in the sequencing run [30].

Raw amplicon sequencing libraries were submitted to the NCBI database under bioproject number PRJNA682516.

### 2.4. Sequence Analysis and 16S rDNA Profiling

Sequence read processing was performed as previously described [30] using the MOTHUR software package v141.1 [31] and VSEARCH algorithm for chimera detection [32]. A clustering distance of 0.03 was used for operational taxonomic unit (OTU) generation. 16S reference alignment and taxonomical assignment from phylum to genus were done with MOTHUR and were based upon the SILVA database (v1.32) of full-length 16S rDNA sequences [33].

Subsample datasets with 8994 cleaned reads per sample were obtained and used to evaluate ecological indicators (Good’s coverage, Chao richness index, reciprocal Simpson microbial diversity, and Simpson derived evenness of the samples) and β-diversity (using a distance Bray–Curtis dissimilarity matrix) using MOTHUR. When assessing the ecology of a community (e.g., microbial), α-diversity measures the diversity within the community as opposed to β-diversity which measures diversity between communities (or the same community at different time points) [34]. Richness is a measure of the number of species in the community and evenness expresses how evenly the individuals in the community are distributed over the different species (e.g., presence of predominant species) [35]. The Good’s coverage estimates what percentage of the total species of a community is represented in a sample.

### 2.5. Data Analysis 

Differences between groups (AM-NS, AM-S, CG) for the different ecological indices of the microbial population were assessed with a non-parametric Kruskal–Wallis test followed by paired post hoc tests corrected with a two-stage linear step-up procedure of Benjamini, Krieger and Yekutieli using PRISM 7 (Graphpad Software; San Diego, CA, USA). Differences were considered significant for a *q*-Value or a *p*-Value < 0.05.

β-diversity was visualized with a Bray–Curtis dissimilarity matrix-based non-parametric dimensional scaling (NMDS) model using vegan (https://cran.r-project.org/web/packages/vegan/index.html (accessed on 11 January 2021)) and vegan3d packages (https://cran.r-project.org/web/packages/vegan3d/index.html (accessed on 11 January 2021)) in R. Sample clustering and beta-dispersion were respectively assessed in a Bray–Curtis dissimilarity matrix with analysis of molecular variance (AMOVA) and homogeneity of molecular variance (HOMOVA) tests using MOTHUR (using 10,000 iterations on the subsampled table). AMOVA determines whether the genetic diversity within two or more communities is greater than their pooled genetic diversity, and HOMOVA determines whether the amount of genetic diversity in each community is significantly different [36]. 

Finally, significant differential bacterial population abundance between groups was performed with two-way ANOVA with a Benjamini–Hochberg false discovery rate correction using the population subsampled table where null median populations were removed.

## 3. Results

### 3.1. AnImals

A total of 85 horses were included in the study. These included 59 horses diagnosed with AM, of which 29 were survivors (AM-S) and 30 were non-survivors (AM-NS), and 26 co-grazers (CG).

The AM-S group included 13 mares, 10 geldings and 6 stallions of a wide variety of breeds (7 Warmbloods, 4 ponies, 3 Haflingers, 3 Arabians, 2 Standardbreds and 10 horses from other breeds) with a median age of 6.9 years (range 0.4 to 25 years). 

The AM-NS group included 13 mares, 13 geldings and 4 stallions of different breeds (including 10 Warmbloods, 5 ponies, 2 Andalusians, 2 Friesians and 11 horses from other breeds) with a median age of 7.5 years (range 0.5 to 22 years). From the 30 non-survivors, 6 died naturally from the disease and 23 were euthanized. Information about the cause of death was missing for one horse. 

Concurrently, feces of 26 co-grazers were collected. The co-grazer group included 9 mares, 4 geldings and one stallion. Different breeds were represented, including 4 Arabians, 4 Warmbloods, 3 ponies and 11 horses from other breeds. The median age of this group was 8.1 years (range 0.6 to 25 years). 

#### Diet

All horses included in the present study had access to pasture, including 35 horses that were living 24 h a day on pasture. Twenty-one horses (14 AM-NS, 7 AM-S) were eating 100% roughage (pasture with or without hay supplement), whereas 11 received some kind of sweet feed supplement on top of roughage or pasture (6 AM-NS, 4 AM-S and 1 CG). Unfortunately, information about diet was unavailable or poorly specified (i.e., lacking amount or type of hay or sweet feed) for 53 of the 85 horses included in this study (10 AM-NS, 18 AM-S and 25 CG).

### 3.2. Analysis of Microbial Population

From 12,025,466 raw sequencing reads, 10,225,098 and 8,353,193 reads were obtained, respectively, after the cleaning process and chimera removal, with a final median read length of 492 nucleotides. Eight thousand nine hundred and ninety-four reads per sample were retained as a subsampling process to proceed with OTU binning (0.03 cut-off) for a total of 23,406 OTUs regrouped into 583 populations at the genus level. Mean Good’s coverage at the genus level was 99.6%, with no statistical difference between groups.

### 3.3. α-Diversity and β-Diversity Analysis

**Figure 1:**
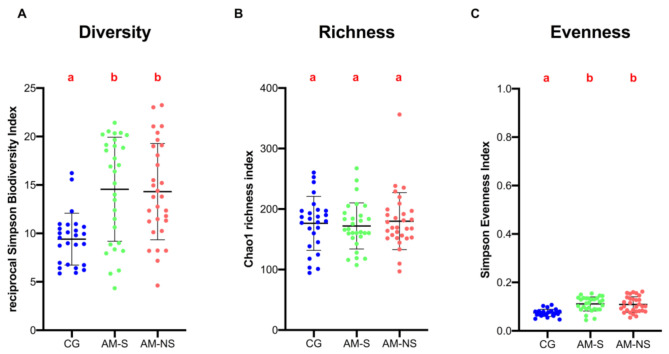
Bacterial intrinsic diversity deduced from inverse Simpson index (**A**). Bacterial genus richness deduced from Chao1 index (**B**). Bacterial genus evenness deduced from Simpson index (**C**). Data are scatter dot plots at the genus level for individual horses in the three defined groups with the mean and standard deviation. Data with different superscript letters are significantly different at *q* < 0.05 (Kruskal–Wallis).

Microbial population ecological indices of fecal samples were assessed at the genus level and are shown in Figure 1.

Results of the Kruskal–Wallis test yielded significant global differences between groups for bacterial alpha-diversity and evenness but not for richness, meaning that the number of bacterial genera present in the feces was not significantly different between groups.

According to results of the paired post hoc test, diversity was significantly higher in horses with AM (survivors and non-survivors) than in CG (*q*-Value < 0.001). No significant differences were found between survivors and non-survivors (*q*-Value = 0.969).

Similarly, genus evenness was found to be significantly higher in horses with AM (survivors and non-survivors) than in CG (*q*-Value < 0.0001). This means that the distribution of bacterial genera (relative abundance) in the feces of horses with AM is more uniform than in the feces of CG, where there is a more marked predominance of certain genera. No significant differences were found between survivors and non-survivors (*q*-Value = 0.616).

**Figure 2:**
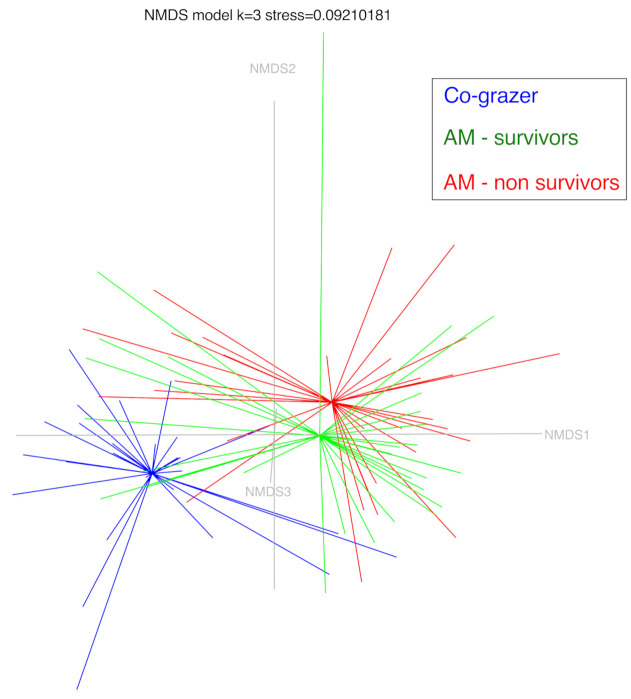
Nonmetric dimensional scaling with three axes of the three horses groups (co-grazers: CG, atypical myopathy survivors: AM-S and atypical myopathy non-survivors: AM-NS). Model stress is 0.092.

β-diversity of the fecal microbial profile was visualized using an NMDS model (three dimensions, stress 0.092) (Figure 2). Group clustering testing (AMOVA) revealed significant differences between CG and horses diagnosed with AM (*p*-Value < 0.00001). No significant differences were found between survivors and non-survivors (*p*-Value = 0.2074). HOMOVA testing yielded significant results, indicating that the amount of population homogeneity in the fecal microbiota was significantly different between CG and horses diagnosed with AM (CG vs. AM-S *p*-Value < 0.00002, CG vs. AM-NS *p*-Value = 0.0001). No significant differences were found between survivors and non-survivors (*p*-Value = 0.8098).

### 3.4. Composition of Fecal Microbiota

**Figure 3:**
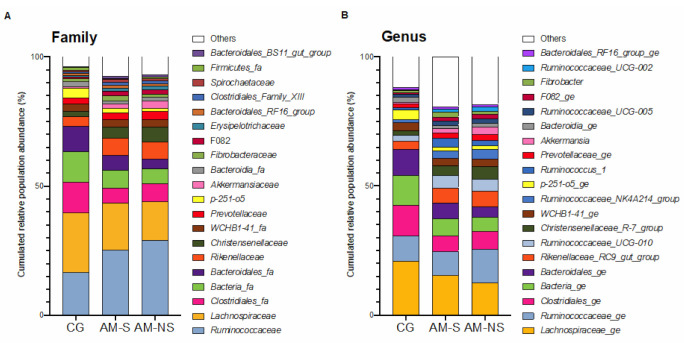
Changes in bacterial populations in the fecal content, assessed by 16S V1-V3 profiling. The bar chart depicts the relative abundance of the bacterial families (**A**) and bacterial genera (**B**) accounting for more than 1% of the total abundance in the feces. Co-grazers (CG) were compared to horses diagnosed with atypical myopathy (AM) with different outcomes (survivors, S, and non-survivors, NS).

A total of 13 different phyla were identified in the feces, the most abundant being Firmicutes and Bacteroidetes. The distribution of the main families and genera and their relative abundance in the three groups of horses is depicted in Figure 3.

At a family level, the most abundant families observed were Ruminococcaceae, Lachnospiraceae, Clostridiales_fa, Bacteria_fa, Bacteroidales_fa, Rikenellaceae, Christensenellaceae, WCHB1-41_fa, Prevotellaceae and p251-o5.

At a genus level, the most abundant defined genera observed were *Lachnospiraceae_ge, Ruminococcaceae_ge, Clostridiales_ge, Bacteria_ge, Bacteroidales_ge, Rikenellaceae_RC9_gut_group, Ruminococcaceae_UCG-010, Christensenellaceae_R-7_group, WCHB1-41_ge, Ruminococcaceae_NK4A214_group, p-251-o5_ge and Ruminococcus_1*.

### 3.5. Statistical Differences in Fecal Microbiota Composition

**Figure 4:**
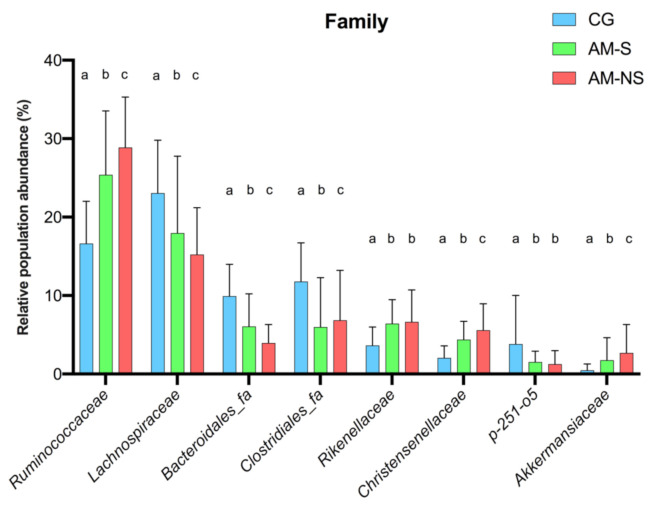
Changes in bacterial family populations in the feces, assessed by 16S V1-V3 profiling and expressed as relative population abundance. Bar plot shows mean value with standard deviation of selected families whose relative abundance is significantly different between the groups (two-way ANOVA with Benjamini–Hochberg false discovery rate correction). Data with different superscript letters are significantly different at *q* < 0.05. Corresponding global *p*-Values and *q*-Values can be found in Table 1. CG: Co-grazers, AM-S: Atypical myopathy survivors, AM-NS: Atypical myopathy non-survivors.

According to global ANOVA results, the relative abundance of eight families differed significantly between horses diagnosed with AM compared to CG: Ruminococcaceae (*p* < 0.0001), Lachnospiraceae (*p* = 0.0053), Clostridiales_fa (*p* = 0.0402), Bacteroidales_fa (*p* < 0.0001), Rikenellaceae (*p* = 0.0062), Christensenellaceae (*p* < 0.0001), p-251-o5 (*p* = 0.0203) and Akkermansiaceae (*p* = 0.0016). A comparison of the relative abundance of these families between the different groups is shown in Figure 4 and Table 1.

Relative abundances of Ruminococcaceae, Lachnospiraceae, Bacteroidales_fa Clostridiales_fa, Christensenellaceae and Akkermansiaceae were significantly different between CG and horses diagnosed with AM, as well as between AM survivors and non-survivors (*p* < 0.05 for all comparisons). Interestingly, the relative abundance of each family increased (Ruminococcaceae, Christensenellaceae, Akkermansiaceae) or decreased (Lachnospiraceae, Bacteroidales_fa, Clostridiales_fa) gradually from CG to non-surviving horses suffering from AM (Figure 4).

Relative abundances of Rikenellaceae and p-251-o5 were lower in CG compared to horses diagnosed with AM (*q*-Value < 0.0001), but no differences were found between AM survivors and non-survivors. Rikenellaceae were less abundant in CG compared to horses with AM (*p* < 0.0001), whereas p-251-o5 were more abundant in CG compared to horses with AM (*p* < 0.0001).

At a genus level, ANOVA global results showed that the relative abundance of 11 genera differed significantly between horses diagnosed with AM compared to those of the CG group: *Lachnospiraceae_ge* (*p* = 0.0009), *Bacteroidales_ge* (*p* = 0.0001), *Christensenellaceae_R-7_group* (*p* = 0.0002), *Ruminococcaceae_NK4A214_group* (*p* < 0.0001), *Ruminococcus_1* (*p* = 0.0080), *Akkermansia* (*p* = 0.0017), *Ruminococcaceae_UCG-002* (*p* = 0.0010), *Rikenellaceae_RC9_gut_group* (*p* = 0.0075), *Ruminococcaceae_UCG-010* (*p* = 0.0011), *p-251-o5_ge* (*p* = 0.0278), *Bacteroidia_ge* (*p* = 0.0026). A comparison of the relative abundance of these genera between the different groups is shown in Figure S1 and Table S1 (Supplementary Data).

Relative abundances of *Lachnospiraceae_ge* and *Bacteroidales_ge* were higher in CG compared to diseased horses, whereas *Christensenellaceae_R-7_group, Ruminococcaceae_NK4A214_group, Ruminococcus_1, Akkermansia* and *Ruminococcaceae_UCG-002* were lower in CG than in horses diagnosed with AM (*p* < 0.05 for all comparisons). Relative abundances of *Lachnospiraceae_ge*, *Bacteroidales_ge* and *Ruminococcus_1* were higher in AM-S compared to AM-NS, whereas *Christensenellaceae_R-7_group, Ruminococcaceae_NK4A214_group, Akkermansia* and *Ruminococcaceae_UCG-002* were lower in AM survivors compared to non-survivors (*p* < 0.05 for all comparisons). Similarly to results at the family level, the relative abundance of most of those genera increased (*Christensenellaceae_R-7_group, Ruminococcaceae_NK4A214_group, Akkermansia* and *Ruminococcaceae_UCG-002*) or decreased (*Lachnospiraceae_ge, Bacteroidales_ge*) gradually from CG to non-surviving horses suffering from AM (Figure S1 in Supplementary Data).

Relative abundances of *Rikenellaceae_RC9_gut_grou, Ruminococcaceae_UCG-010* and *p-251-o5_ge* were significantly different between CG and horses diagnosed with AM (*p* < 0.05 for all comparisons), but no differences were found between AM survivors and non-survivors. *Rikenellaceae_RC9_gut_grou* and *Ruminococcaceae_UCG-010* were significantly less abundant in CG in regard to horses with AM, whereas *p-251-o5_ge* were significantly more abundant in CG in regard to horses with AM (*p* < 0.05 for all comparisons).

## 4. Discussion

This study demonstrates significant differences in the structure and composition of fecal microbiota in horses with AM in regard to their healthy co-grazers. Diversity was significantly higher in horses with AM (survivors and non-survivors) and relative abundances of certain bacterial families were significantly higher (Ruminococcaceae, Christensenellaceae, Rikenellaceae and Akkermansiaceae) or lower (Lachnospiraceae, Clostridiales_fa, Bacteroidales_fa, p-251-o5) than in co-grazers. Interestingly, most of these bacterial shifts were significantly more pronounced in non-survivors.

When interpreting microbiome changes associated with a specific disease, two hypotheses arise: changes can be either a trigger or a consequence of the disease. A specific microbiome (or its metabolome) can be a predisposing/protecting factor for the disease, providing each individual with a major or minor susceptibility to the disease. On the other hand, the disease process itself can induce more or less profound changes in the microbiome of an individual. Untangling the cause–consequence relationship between the intestinal microbiome and AM in horses might aid in developing therapeutic and/or preventive strategies for at-risk horses.

### 4.1. Differences in Community Structure between CG and Horses with AM

Fecal bacterial richness did not appear to be affected by clinical disease in the present study, but diversity and evenness were significantly higher in horses with AM than in CG.

Diseases characterized by bacterial dysbiosis in horses, such as colitis, have been associated with lower diversity, lower richness and lower evenness in fecal microbiota in regard to healthy horses [37,38]. Changes in fecal bacterial diversity have also been observed to be associated with other equine disorders, such as equine metabolic syndrome (EMS). Horses with EMS showed lower fecal microbial diversity when compared to controls in one study [19], whereas horses suffering from naturally occurring chronic laminitis showed higher fecal microbial diversity compared to non-laminitic control horses [39]. In vitro and in vivo studies have demonstrated a causative link of carbohydrate-induced laminitis and alterations in the gut microbiome [18,40,41,42], however, the relevance of fecal bacterial diversity changes concerning horses with EMS or AM remains to be determined.

Similarly to humans [43], one study reported a reduction in fecal microbial diversity in aged animals (19 to 25 years old) in regard to adult horses (5 to 12 years old), although no significant differences in specific bacterial populations were found [44]. In the present study, the median and range of age of the three groups of horses compared were quite similar, so a bias of age affecting the results is unlikely.

Taking into account that horses diagnosed with AM were treated in referral hospitals, and most co-grazers were sampled at the barn, transport to the hospital could have interfered with disease-associated results. A recent study found that transportation of healthy horses to a hospital facility resulted in an increase in richness and Shannon diversity index which normalized within 24 h [45]. On the contrary, a previous study did not find any effect of transport on alpha-diversity indices in adult horses after 1h of transport (richness, diversity, evenness) [46]. In the present study, the vast majority of diseased horses had a transport time to the hospital ranging from 30 min to 2 h.

This is, to our knowledge, the first study assessing microbiota in horses with AM [7,47]. The interpretation of increased diversity and evenness in the AM group is not clear to date. The effect of HGA or MCPG on fecal microbiota has never been studied in the equine species, so it remains unknown if protoxins (or their toxic metabolites) could alter the structure or composition of fecal microbiota in horses ingesting them. 

### 4.2. Differences in Community Composition Between CG and Horses with AM

The predominant phyla in the three groups of this study were Firmicutes and Bacteroidetes. Bacterial shifts observed between horses suffering from AM and healthy co-grazers affected mainly families and genera included in the core equine intestinal microbiota [17,48,49,50]. The term “core microbiota” refers to the key bacterial populations which are present in most individuals, probably playing essential roles and defining what is considered as a “healthy microbiota” [51].

Lachnospiraceae and Ruminococcaceae are members of the Firmicutes phylum and constitute two of the main families of active bacteria present in the distal gut of both humans and horses [52,53]. Both families play important roles in gastrointestinal health: they are cellulolytic, fibrolytic bacteria, and major producers of short-chain fatty acids [51]. Some of the species in these families produce butyrate, which has been related to a healthy colonic mucosa [54]. Several studies have shown consistently lower relative abundances of Lachnospiraceae and Ruminococcaceae families in humans and animals with gastrointestinal disease [13,17,37].

Evidence in human and equine medicine shows that diet, particularly dietary carbohydrates, can influence the composition and stability of the gut microbiome [44,55,56,57,58]. A study found that the relative abundances of Lachnospiraceae and Bacteroidetes were significantly greater and Ruminococcaceae significantly lower in concentrate-fed horses compared with grass-fed horses [59]. All horses included in the present study had access to pasture, including 35 horses that were living 24 h a day on pasture. Twenty-one horses were eating 100% roughage (pasture with or without hay supplement), whereas 11 received some kind of sweet feed supplement on top of roughage or pasture. Unfortunately, information about diet was unavailable or poorly specified (i.e., lacking amount or type of hay or sweet feed) for 53 of the 85 horses included in this study, which makes it difficult to interpret and discuss the results in relation to diet. However, as the CG horses were kept on the same pastures as the AM-affected horses, they most likely received the same diet as the diseased horses, as grazing horses are very rarely fed individually.

The Lachnospiraceae family is the main component of a select core bacterial community which is found in all horses regardless of age or diet, and some authors suggest that it could explain why horses are especially susceptible to metabolic dysfunction [44]. Members of this family seem to play a protective role against disease in humans and animals. It has been suggested that the indigenous microbiota could inhibit the colonization of pathogenic bacteria by several mechanisms, including the production of metabolites such as short-chain fatty acids, bacteriocins or other antimicrobial compounds, as well as competing for limiting nutrients (nutrient niche hypothesis) [60]. Many members of the family Lachnospiraceae are relevant for the growth of other microbes and host epithelial cells due to their production of butyric acid. Based on this ability, Lachnospiraceae have been associated with protection from colon cancer in humans by activating the activating protein 1 (AP-1) signaling pathway in human epithelial cells [61,62,63]. This pathway, among others, is involved in cellular growth control, and therefore regulates cell life or death in connection with extracellular stimuli [64]. If the same mechanism happens in equine intestinal epithelial cells, a decrease in Lachnospiraceae might impact their proliferation and likely affect the mucosal barrier function, making it easier for undesired gut contents, like toxins, to trespass into the circulation. However, more studies are needed to confirm this link, especially in horses. As horses with AM seem to have a decrease in the relative abundance of Lachnospiraceae, it can be hypothesized that the aforementioned protective roles of this family are diminished in horses suffering from the disease, which could potentially favor disease progress. Further investigation of this link might aid in discovering preventative or treatment strategies against AM.

The family Christensenellaceae, within the bacterial phylum Firmicutes, has gained increasing recognition in recent years due to its strong link to human body condition (BC), as shown in multiple studies investigating different populations. An inverse relationship between host body mass index (BMI) and the relative abundance of Christenellaceae has been demonstrated in humans, and an adaptation of the relative abundance of this family has been observed in fecal microbiota when obese individuals are losing weight [65,66,67]. Interestingly, a previous study demonstrated that lean horses were more prone to developing clinical signs of AM [3]. However, to the authors’ knowledge, a correlation between BC and the relative abundance of Christenellaceae has not yet been demonstrated in horses, but it could be interesting to evaluate. Body condition was not adequately referenced for all horses included in this study but available information from hospital files revealed an average BC for most diseased horses (4–5/9 Henneke BC score), with no cases of extreme obesity or emaciation.

Evidence concerning the relation between the microbiota composition and BC in horses is conflicting to date [44,68,69]. Similarly to the present results, a study in horses with EMS found an increase in the family Akkermansiaceae (phylum Verrucomicrobia) in regard to control horses [19]. The genus *Akkermansia*, a group of mucolytic mucosa-associated bacteria, has received special attention in the literature as a biomarker for a healthy intestine as its relative abundance is inversely correlated to human intestinal disease, such as inflammatory bowel disease, Crohn’s disease, ulcerative colitis and appendicitis, but also extra-intestinal issues such as obesity and autism in children [70,71,72,73,74,75]. Interestingly, those bacteria seem to thrive following decreased intestinal nutrient availability, as seen in intestinal contents of human patients after gastric bypass surgery or the cecum of hamsters that are fasted [73,76]. Oral administration of *Akkermansia* *spp.* has been shown to improve glucose tolerance in obese mice [77]. Such effects could be interesting in a situation of impaired lipid metabolism but preserved glycolytic pathways, as is the case for AM-affected horses [78].

Whether these bacterial shifts observed in horses with AM when compared to co-grazers are a consequence of the intoxication or occurred prior to ingestion of the protoxin remains to be determined. Prospective studies following exposure to the toxin and its consequences on fecal microbiota in horses grazing in at-risk pastures could help to answer that question.

### 4.3. Association with Outcome in Horses with AM

Most bacterial shifts observed in this study between horses suffering from AM and healthy co-grazers were significantly more pronounced in non-survivors than in survivors. This gradual increase/decrease in certain bacterial populations in the feces of horses with AM associated with severity of the disease could suggest a dysbiosis that worsens as the disease progresses. The significance of this finding is unclear, and the nature of this correlation remains to be determined. but may be related to the amount of toxin ingested, which could be higher in non-survivors. Because the study was conducted in naturally occurring cases of AM, the exact amount of HGA and MCPG ingested by each horse remains undetermined, and this factor might have influenced morbidity and mortality. However, the serum acylcarnitine profile (Table S2) of non-surviving horses in this study showed more severe changes than in survivors, suggesting worse metabolic imbalances in horses with a poor outcome. The serum acylcarnitine profile has been shown to be a good predictor for survival in horses with AM [29].

Considering the potential of some bacteria to metabolize amino acids, another hypothesis could be that intestinal microbiota might contribute to the conversion of HGA into MCPA-CoA, accelerating the intoxication process. A wide range of bacteria, including the genera *Fusobacterium, Bacteroides, Propionibacterium, Actinomyces, Peptococcus, Streptococcus, Ruminococcus, Megasphaera* and even *Enterobacteria* (*Escherichia coli, Klebsiella sp., Streptococcus sp*.), have been reported as being responsible for amino acid metabolism in the gut [24,79]. Whether the significantly increased relative abundance of Ruminococcaceae observed in this study in horses suffering from AM is related to this latter hypothesis remains to be determined and deserves further study.

### 4.4. Limitations

This study has certain limitations. Single samples, in general, provide more limited information than a multiple sample follow-up. The results are based on fecal microbiota, which is acceptable to represent the distal part of the equine digestive system, but cannot be used to predict microbiota of the proximal digestive tract [48,80]. Whether the microbiota of the proximal intestinal tract is altered in horses with AM and how this affects progression of the disease remain to be studied. Furthermore, only the bacterial populations in the feces were analyzed, leaving out the small but significant groups of eukaryotes, archaea, viruses and fungi that are equally present in the gastrointestinal tract [81].

Dietary management is known to affect the composition of fecal microbiota and diet was not homogeneous in this study (i.e., some horses received sweet feed supplement whereas other received only roughage). Unfortunately, a lot of detail on dietary data was missing, which made it difficult to analyze, interpret and discuss the results in regard to diet groups. It should be noted that not all horses included in the study had a corresponding co-grazer in the CG group. Some horses were living alone and no companions were available, sometimes all horses in the same pasture were sick or management of co-grazers from a sick case had already been modified by the owner (e.g., removal from pasture, dietary supplementation) before the 24 h window.

Some of the horses in our study were humanely euthanized rather than dying naturally from the disease, which might have created a bias in the AM-NS group and therefore influenced our results regarding disease outcome. On the other hand, the severity of clinical signs in horses that were euthanized was clearly more pronounced compared to clinical signs of survivors, which in most cases ultimately led to the decision to euthanize the animal.

Finally, diseased horses included in this study were referred cases and sometimes the field practitioner had administered some medication prior to referral (i.e., charcoal or paraffin oil, non-steroidal anti-inflammatory drugs and/or vitamins) which could have influenced the results of this study. Fecal sampling in sick horses was performed on admission to the hospital, and in most cases within the first 24 h of clinical manifestation of the disease. In consequence, it seems very unlikely that any treatment administered by the veterinarian may have influenced the composition of fecal microbiota in these horses.

## 5. Conclusions

This study demonstrates significant differences in the structure and composition of the fecal microbiota of AM-affected horses when compared to their non-affected co-grazers. The families Ruminococcaceae, Christensenellaceae and Akkermansiaceae increased, whereas the Lachnospiraceae, Bacteroidales and Clostridiales decreased in horses with AM, especially in those with a poor prognosis. Further studies are needed to determine if those changes precede the disease or are a consequence of the intoxication. Either way, the findings of this study constitute a first step in understanding the possible role of intestinal microbiota in AM and may open new fields for the prevention and treatment of this devastating disease.

## Figures and Tables

**Table 1:** *q*-Values corresponding to comparisons between groups (two-way ANOVA with Benjamini–Hochberg false discovery rate correction) of the eight families depicted in Figure 4. CG: Co-grazers, AM-S: Atypical myopathy survivors, AM-NS: Atypical myopathy non-survivors.

Family	Global *p*-Values (Corrected)	CG vs. AM-S	CG vs. AM-NS	AM-S vs. AM-NS
Ruminococcaceae	<0.0001	**<0.0001**	**<0.0001**	**<0.0001**
Lachnospiraceae	0.0053	**<0.0001**	**<0.0001**	**<0.0001**
Bacteroidales_fa	<0.0001	**<0.0001**	**<0.0001**	**<0.0001**
Clostridiales_fa	0.0402	**<0.0001**	**<0.0001**	**0.0331**
Rikenellaceae	0.0062	**<0.0001**	**<0.0001**	0.2263
Christensenellaceae	<0.0001	**<0.0001**	**<0.0001**	**0.0176**
p-251-o5	0.0203	**<0.0001**	**<0.0001**	0.2147
Akkermansiaceae	0.0016	**0.0067**	**<0.0001**	**0.0249**

Bold values represent *q*-Value < 0.05.

## Data Availability

Publicly available datasets were analyzed in this study. This data can be found here: NCBI database, bioproject number PRJNA682516.

## Supplementary Materials

The following are available online at https://www.mdpi.com/2076-2615/11/2/506/s1. Figure S1: Changes in bacterial genus populations in the feces assessed by 16S V1-V3 profiling and expressed as relative population abundance, Table S1: *q*-Values corresponding to comparisons between groups (two-way ANOVA with Benjamini–Hochberg false discovery rate correction) of the 11 genera depicted in Figure S1. Table S2: Serum acylcarnitine concentrations from horses with AM included in this study.
