# Supplementary material for: Comparison of Fecal Microbiota of Horses Suffering from Atypical Myopathy and Healthy Co-Grazers
Source: Animals (Basel). 2021 Feb 15;11(2):506. doi: 10.3390/ani11020506 (PMC7919468; doi:10.3390/ani11020506)
Supplement: Supplementary file 1 [file animals-11-00506-s001.pdf]

animals

MDPI

Supplementary material

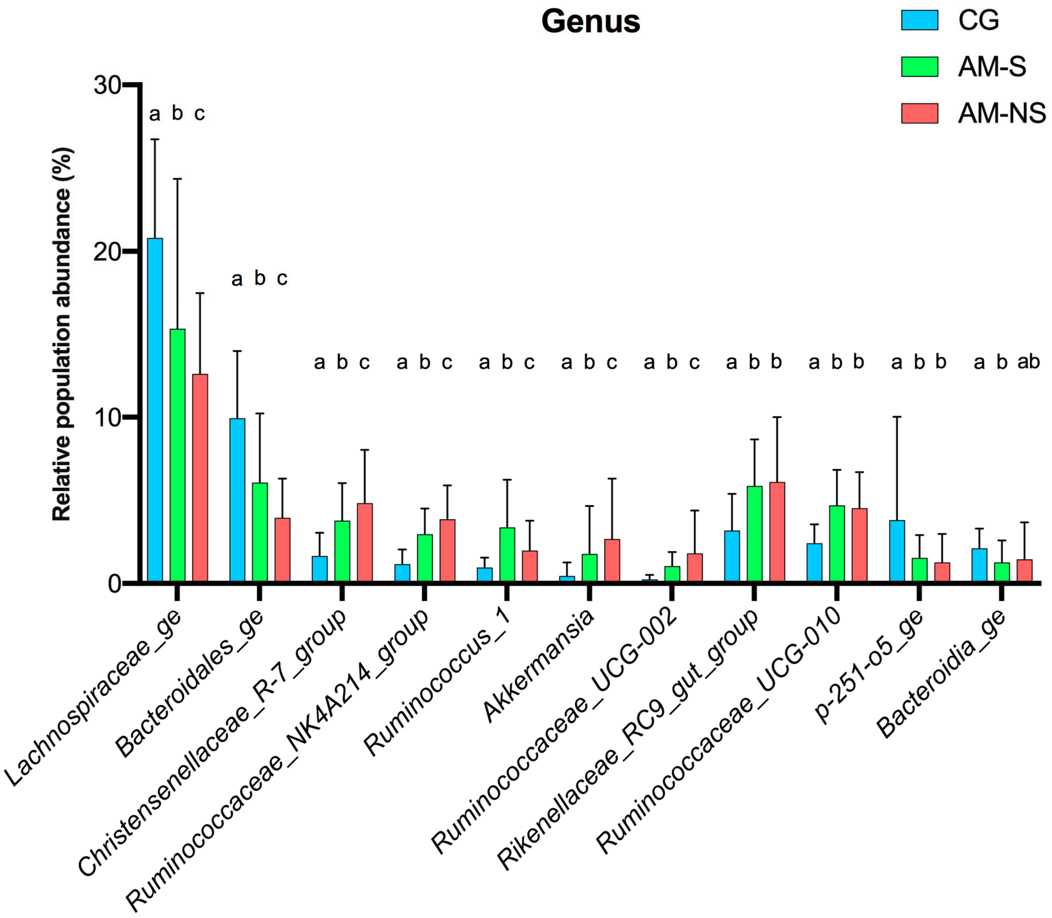

Figure S1. Changes in bacterial genus populations in the feces assessed by 16S V1-V3 profiling and expressed in relative population abundance. Bar plot showing mean value with standard deviation of selected genera whose relative abundance is significantly different between the groups (2 way ANOVA with Benjamini-Hochberg's false discovery rate correction). Data with different superscript letters are significantly different at $q < 0.05$. Corresponding global p-values and q-values can be found in Table S1. CG: Co-grazers, AM-S: Atypical Myopathy-Survivor, AM-NS: Atypical Myopathy-Non-Survivors.

Table S1. q-values corresponding to comparisons between groups (2 way ANOVA with Benjamini-Hochberg's false discovery rate correction) of the 11 genera depicted in Figure S1. CG: Co-grazers, AM-S: Atypical Myopathy-Survivor, AM-NS: Atypical Myopathy-Non-Survivors.

**Genus**	**Global p-values (corrected)**	**CG vs AM-S**	**CG vs AM-NS**	**AM-S vs AM-NS**
Lachnospiraceae_ge	0.0009	**<0.0001**	**<0.0001**	**<0.0001**
Bacteroidales_ge	0.0001	**<0.0001**	**<0.0001**	**<0.0001**
Christensenellaceae_R-7_group	0.0002	**<0.0001**	**<0.0001**	**0.0023**
Ruminococcaceae_NK4A214_group	<0.0001	**<0.0001**	**<0.0001**	**0.0083**
Ruminococcus_1	0.0080	**<0.0001**	**0.0046**	**<0.0001**
Akkermansia	0.0017	**0.0004**	**<0.0001**	**0.0090**
Ruminococcaceae_UCG-002	0.0010	**0.0314**	**<0.0001**	**0.0314**
Rikenellaceae_RC9_gut_group	0.0075	**<0.0001**	**<0.0001**	0.1596
Ruminococcaceae_UCG-010	0.0011	**<0.0001**	**<0.0001**	0.2240
p-251-o5_ge	0.0278	**<0.0001**	**<0.0001**	0.1616
Bacteroidia_ge	0.0026	**0.0332**	**0.0649**	0.3928

Bold values represent *q*-Value < 0.05.

Table S2. Serum concentrations (median, maximal and minimal values) of free carnitine and acylcarnitines (µmol/L) from horses with atypical myopathy included in this study. AM-S: Atypical Myopathy-Survivors, AM-NS: Atypical Myopathy-Non-Survivors.

			AM-S			AM-NS	
Group	Variable	Min	Median	Max	Min	Median	Max
Free Carnitine	Free Carnitine	11.16	49.62	378.79	23.78	104.41	392.11
Short chain AC (C2 to C5)	C3DC-Carnitine	0.03	0.12	0.95	0.07	0.33	1.60
	C5DC-Carnitine	0.24	1.14	2.95	0.45	2.23	9.42
	C2-Carnitine	6.31	25.59	82.79	3.63	39.57	123.52
	C3-Carnitine	0.63	1.44	5.47	0.40	2.95	6.91
	C4-Carnitine	1.22	10.98	47.72	1.84	21.81	60.31
	C5-Carnitine	1.41	6.13	30.85	4.25	22.08	49.62
	C5-OH-Carnitine	0.10	0.24	1.21	0.16	0.64	1.90
Medium chain AC (C6 to C10)	C8-Carnitine	0.14	1.21	5.10	0.20	1.77	14.75
	C10:2-Carnitine	0.06	0.56	2.78	0.08	0.71	6.73
	C10:1-Carnitine	0.03	0.37	1.28	0.07	0.36	2.43
	C10-Carnitine	0.05	0.42	1.91	0.10	0.46	4.68
	C6-Carnitine	0.18	2.14	15.60	0.52	4.03	28.22
	C8:1-Carnitine	0.07	0.57	5.16	0.18	1.00	5.98

Long chain AC (C12 to C18)	C12:1-Carnitine	0.03	0.15	0.54	0.05	0.22	0.85
	C12-Carnitine	0.05	0.21	0.65	0.09	0.25	1.35
	C14:1-Carnitine	0.03	0.26	0.98	0.11	0.27	1.58
	C14-Carnitine	0.04	0.20	0.42	0.08	0.23	0.92
	C16:1-Carnitine	0.04	0.17	0.50	0.09	0.22	1.74
	C16-Carnitine	0.20	0.51	2.41	0.28	0.65	3.05
	C18:1-Carnitine	0.15	0.46	1.55	0.21	0.61	2.80
	C18-Carnitine	0.09	0.19	0.75	0.10	0.32	1.07
